# Observer’s anxiety facilitates magnocellular processing of clear facial threat cues, but impairs parvocellular processing of ambiguous facial threat cues

**DOI:** 10.1038/s41598-017-15495-2

**Published:** 2017-11-09

**Authors:** Hee Yeon Im, Reginald B. Adams, Jasmine Boshyan, Noreen Ward, Cody A. Cushing, Kestutis Kveraga

**Affiliations:** 1 Athinoula A. Martinos Center, Department of Radiology, Massachusetts General Hospital, Charlestown, MA USA; 2 Department of Radiology, Harvard Medical School, Boston, MA USA; 3 Department of Psychology, The Pennsylvania State University, State College, PA USA

## Abstract

Facial expression and eye gaze provide a shared signal about threats. While a fear expression with averted gaze clearly points to the source of threat, direct-gaze fear renders the source of threat ambiguous. Separable routes have been proposed to mediate these processes, with preferential attunement of the magnocellular (M) pathway to clear threat, and of the parvocellular (P) pathway to threat ambiguity. Here we investigated how observers’ trait anxiety modulates M- and P-pathway processing of clear and ambiguous threat cues. We scanned subjects (N = 108) widely ranging in trait anxiety while they viewed fearful or neutral faces with averted or directed gaze, with the luminance and color of face stimuli calibrated to selectively engage M- or P-pathways. Higher anxiety facilitated processing of clear threat projected to M-pathway, but impaired perception of ambiguous threat projected to P-pathway. Increased right amygdala reactivity was associated with higher anxiety for M-biased averted-gaze fear, while increased left amygdala reactivity was associated with higher anxiety for P-biased, direct-gaze fear. This lateralization was more pronounced with higher anxiety. Our findings suggest that trait anxiety differentially affects perception of clear (averted-gaze fear) and ambiguous (direct-gaze fear) facial threat cues via selective engagement of M and P pathways and lateralized amygdala reactivity.

## Introduction

Facial expression and direction of eye gaze are two important sources of social information. Reading emotional expression can be informative in understanding and forecasting an expresser’s behavioral intentions^1–5^, and understanding eye gaze allows an observer to orient spatial attention in the direction signaled by the gaze^6–11^. Individuals with many cognitive disorders show distorted ability in perceiving social cues from faces (e.g., generalized anxiety disorder^12,13^, social anxiety disorder^14,15^, and depression^16^ or impaired (e.g., prosopagnosia^17,18^ and autism^19,20^).

The signals that facial expression and eye gaze convey interact in the perceiver’s mind. A fearful facial expression with an averted gaze is typically recognized as indicating a threat located where the face is “pointing with the eyes”^21^. Both fear and averted gaze are avoidance-oriented signals and together represent congruent cues of threat, where the eye gaze direction indicates the potential location of the threat causing the fear in the expresser^22–26^. Conversely, direct gaze is an approach-oriented cue directed at the observer, while fear is an avoidance cue. Thus, unless the observer is the source of threat, fear with direct gaze is a more ambiguous combination of threat cues because it is unclear whether the expresser is signaling danger or attempting to evoke empathy^21^.

Consistent with these interpretations, observers tend to perceive averted-gaze fear (clear threat) as more intense and recognize it more quickly and accurately compared to direct-gaze fear [^1,24–28^, ambiguous threat]. Eye gaze also influences the perception of emotion in neutral faces: Approach-oriented emotions (anger and happy) are attributed to neutral faces posed with direct gaze whereas avoidant-oriented emotions (fear and sadness) are attributed to neutral faces posed with averted gaze^25,29^.

Neuroimaging studies have highlighted the role of the amygdala in such integration of emotional expression with eye gaze^21,23,25,26,30–36^. The amygdala reactivity to the interaction of facial expression with eye gaze has been shown to be modulated by presentation speed^23,30,33^ and this modulation differs by hemisphere^23,30,33,37^. These findings suggest that the bilateral amygdalae are differentially involved in processing of clear, congruent threat cues (averted-gaze fear) and ambiguous threat cues (direct-gaze fear). Specifically, left and right amygdalae showed heightened activation to longer exposures of direct-gaze fearful faces (ambiguous threat), and to shorter exposures of averted-gaze fearful faces (clear threat), respectively^23,30,33^. This suggests that the right amygdala may be more involved in early detection of clear threat cues and the left amygdala may engage more in considered assessment and evaluation of ambiguous threat cues.

According to dual process models^38–41^, “reflexive” and “reflective” processes operate as a fast, automatic response, and a relatively effortful, top-down controlled process for finer-tuned information processing, respectively. The dual process model also has a direct parallel in the threat perception literature, which has proposed the existence of the so-called “low road” vs. “high road” routes^42–44^. The “low-road” is considered to be an evolutionarily older pathway (presumably via the coarser, achromatic magnocellular (M) pathway projections), for processing of “gist” and rapid defensive responses to threat without conscious thought^42,45–47^. The “high-road”, on the other hand, is considered as the route for a slower, conscious processing of detailed information, allowing for modulation of initial low-road processing^42,43,45^ and might be subserved predominantly by the parvocellular (P) pathway projection through the ventral temporal lobe.

Current models of face perception also support similar, parallel pathways in the human visual system (M and P) that are potentially tuned to different processing demands. For example, Vuilleumier *et al*.^48^ suggested that low spatial frequency (LSF) and high spatial frequency (HSF) information of fearful face stimuli are carried in parallel via M- and P-pathways, respectively. By exploiting face stimuli designed to selectively bias processing toward M- vs. P-pathway, Adams *et al*.^31^ recently found brain activations for averted-gaze fear faces (clear threat) in M-pathway regions and for direct-gaze fear faces (ambiguous threat) in P-pathway regions. Such dissociation in the responsivity of the M- and P-pathways to clear and to ambiguous threat signals, respectively, led the authors to suggest that fearful face by eye gaze interaction may engage a similar, generalizable dual process of threat perception that engages reflexive (via M-pathway) and reflective (via P- pathway) responses^23,31^.

Although substantial individual differences in behavioral responses and amygdala reactivity to emotional faces have been closely associated with observers’ anxiety^13,49–54^, only a few studies have systematically examined the role of anxiety in such integrative processing of facial fear and eye gaze^11,29,55–57^. They found that high trait anxiety individuals show more integrative processing of facial expressions and eye gaze for visual attention^11^, stronger cueing effect by eye gaze in fearful expressions^56,57^, and increased amygdala reactivity to compound threat-gaze cues^29^, suggesting that high anxiety level is associated with higher sensitivity and stronger reactivity to compound threat cues in general. To our knowledge, none of the studies has examined how observer anxiety interacts with the bilateral amygdala reactivity during perception of facial threat cues via M vs. P visual pathways. Thus, better understanding of how perceivers’ anxiety modulates the processing of facial threat cues via M and P visual pathways would contribute to the refinement of behavioral and neuroanatomic models for anxiety-related disorders.

The primary aim of the current study was to examine how perceiver’s anxiety modulates behavioral and neural responses to averted-gaze fear (clear threat) vs. direct-gaze fear (ambiguous threat) in a larger (N of 108) and more representative cohort, compared to previous studies that exploited relatively modest sample sizes (e.g., N = 27^49^, N = 31^29^, and N = 32^54^). Based on the recent findings^31^ that M- and P-pathways differentially favor compound threat cues depending on the clarity or ambiguity of threat, we hypothesized that the association between high anxiety and increased amygdala reactivity would be observed specifically for averted-gaze fear faces (e.g., clear threat) projected to M-pathway, and for direct-gaze fear (e.g., ambiguous threat) projected to P-pathway. To test our hypothesis, we created our face stimuli that were designed to selectively engage M or P processing by calibrating their luminance and color for each individual in separate procedures immediately before commencing the experiment, as in the previous studies^58–61^. We then examined participants’ behavioral and amygdala responses to clear threat cues (averted-gaze fear faces) presented in the low-luminance, grayscale M-biased image and to ambiguous threat cues (direct-gaze fear faces) presented in the isolumance, chromatic (red-green) P-biased image.

Furthermore, we also wanted to examine how anxiety-related modulation of neural activity varies between the hemispheres, given the previous evidence for hemispheric difference in the amygdala activity in perception of facial fear or anger^23,31,33,62,63^. More right amygdala involvement was observed in responding to subliminal threat stimuli^63^ and briefly-presented, averted-gaze fear faces (clear threat cues:^23,33^) or averted-gaze eyes^62^; whereas more left amygdala involvement was observed in responding to supraliminal threat stimuli^63^ and ambiguous threat cues conveyed by direct-gaze fear faces^23,31,62^. Therefore, another aim of our study was to directly test laterality effects in the activation pattern of the left and right amygdala in response to facial fear and their modulation by perceiver anxiety. Based on the previous findings and proposed framework^23,31,33,42,43,45,48^, we specifically predicted that observers’ anxiety would differentially modulate the right amygdala reactivity to clear threat cues (averted-gaze fear faces) in the M-biased stimuli and the left amygdala reactivity to ambiguous threat cues (direct-gaze fear faces) in the P-biased stimuli.

## Results

**Figure 1:**
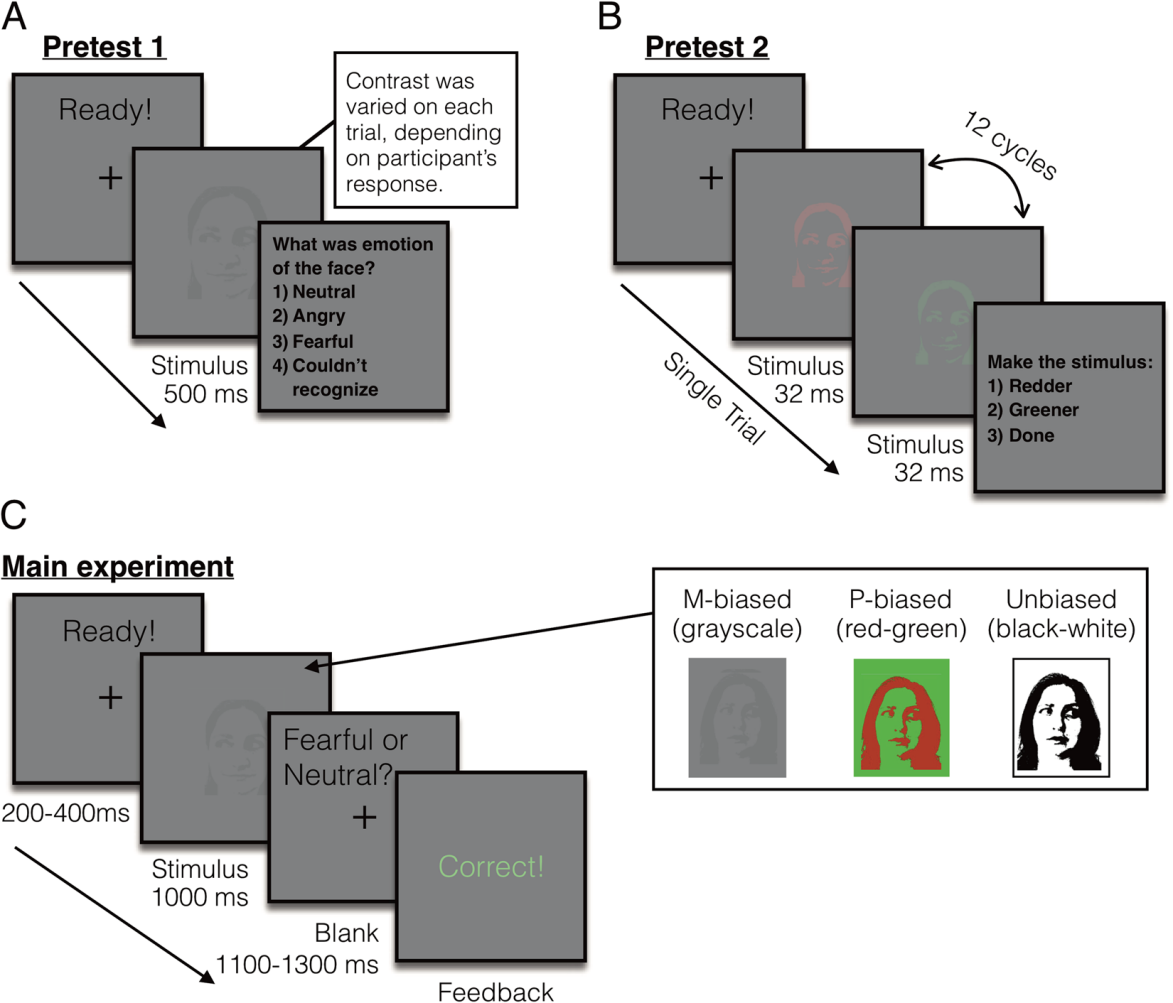
Sample trials of the pretests and the main experiment. (**A**) A sample trial of pretest 1 to measure the participants’ threshold for the foreground-background luminance contrast for achromatic M-biased stimuli. (**B**) A sample trial of pretest 2 to measure the participants’ threshold for the isoluminance values for chromatic P-biased stimuli. (**C**) A sample trial of the main experiment and sample images of M-biased (grayscale), P-biased (red-green), and Unbiased (black-white) stimuli.

Participants (N = 108) viewed images of fearful or neutral faces with direct and averted gaze with one-second presentations while undergoing fMRI. The stimuli were two-tone images of faces presented as high-luminance contrast (Unbiased), low-luminance contrast (M-biased), or isoluminant red/green, chromatically defined (P-biased) images (examples are shown in Fig. 1C). Participants were asked to report whether the face presented in the stimulus looked fearful or neutral. In order to investigate the effects of anxiety on M-pathway processing of clear threat cues and P-pathway processing of ambiguous threat cues, we examined the relationship between participants’ anxiety and behavioral measurements (accuracy and RT), and amygdala activation during perception of the stimuli containing different facial expressions and eye gaze in M- and P-biased images. We used trait anxiety rather than state anxiety to investigate the more enduring effects of anxiety^11^. Participants’ state anxiety scores ranged from 20 to 64 (mean = 32.6, SD = 9.4), and trait anxiety scores from 20 to 66 (mean = 33.9, SD = 9.7). The participants’ trait anxiety highly correlated with their state anxiety scores (*r* = 0.71, *p* < 0.001).

The beta estimates extracted from the amygdala and the behavioral measurements were screened for outliers (3 SD above the group mean) within each condition. As a result, 1.62% and 1.16% of the data points on average were excluded from response time (RT) and from accuracy, and 0.69% and 0.93% of the data points on average were excluded from the left and right amygdala activation for the further analyses. We report here the partial correlation coefficient *r* after controlling for the effects of the participants’ age and sex.

### Behavioral results

**Figure 2:**
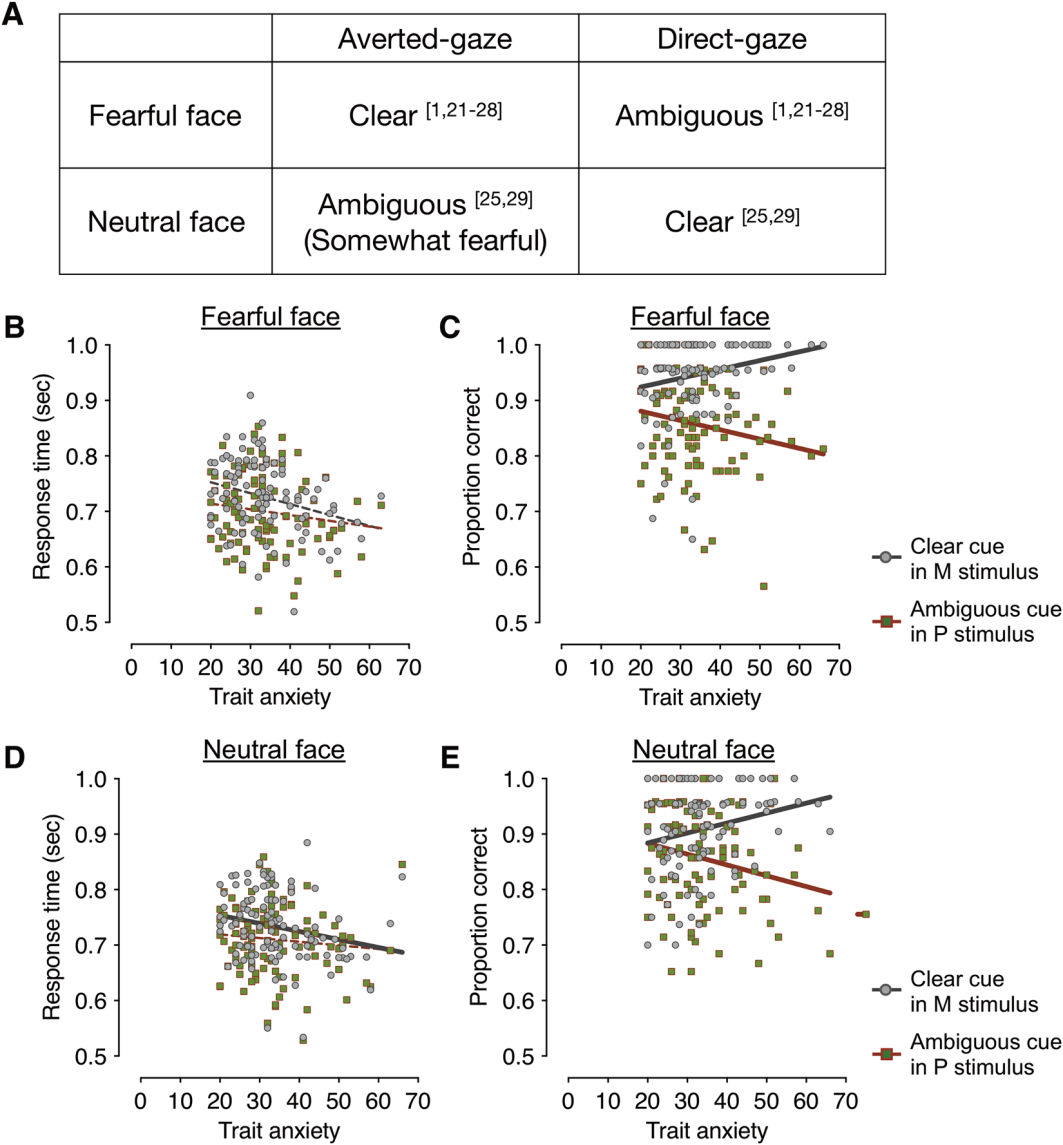
The behavioral results from the main experiment. (**A**) Different combinations of facial expressions (fearful and neutral) and eye gaze (averted and direct) that convey clear and ambiguous cues. Numerical superscripts indicate relevant references. (**B**) The response time (RT) for clear fear (averted-gaze fear) faces presented in M-biased (dots in gray) and for ambiguous fear (direct-gaze fear) faces presented in P-biased (dots in red-green) stimuli. The gray and red lines indicate the linear relationship between trait anxiety and the RT for the M-biased stimuli and the P-biased stimuli, respectively. Solid, thicker lines indicate significant correlations (FDR adjusted *p* < 0.05), whereas broken, thinner lines indicate correlations that were not statistically significant. (**C**) The accuracy for clear fear (averted-gaze fear) faces presented in M-biased (dots in gray) and for ambiguous fear (direct-gaze fear) faces presented in P-biased (dots in red-green) stimuli. Solid, thicker lines indicate significant correlations (FDR adjusted *p* < 0.05), whereas broken, thinner lines indicate correlations that were not statistically significant. (**D**) The response time (RT) for clear neutral (direct-gaze neutral) faces presented in M-biased (dots in gray) and for ambiguous neutral (averted-gaze neutral) faces presented in P-biased (dots in red-green) stimuli. Note that the type of eye gaze that is combined to neutral face for clear cue is different from fearful face. (**E**) The accuracy for clear neutral (direct-gaze neutral) faces presented in M-biased and for ambiguous neutral (averted-gaze neutral) faces presented in P-biased stimuli.

Figure 2B and C show the mean response times (RT) of the correct trials and mean accuracy as a function of trait anxiety scores, when participants viewed the averted-gaze fear (e.g., clear threat; Fig. 2A) presented in the M-biased stimulus (grayscale image) and the direct-gaze fear (ambiguous threat; Fig. 2A) in the P-biased stimulus (red-green image). As shown in Fig. 2B, the correlation between the participants’ anxiety scores and the RT for averted-gaze fear (clear threat cue) presented in M-biased stimulus was not statistically significant, after regressing out the participants’ age and sex (*r* = −0.175, FDR adjusted *p* = 0.140). The median RT showed similar patterns: *r* = −0.187, FDR adjusted *p* = 0.108. Likewise, neither the mean RT nor the median RT for direct-gaze fear (ambiguous threat cue) presented in P-biased stimulus showed significant correlation with the trait anxiety (mean RT: *r* = −0.106, FDR adjusted *p* = 0.281; median RT: *r* = −0.106, FDR adjusted *p* = 0.278).

Unlike the RT, however, participants’ accuracy significantly correlated with their anxiety scores: As shown in Fig. 2C, participants with higher-trait anxiety were more accurate in recognizing averted-gaze fear (clear threat cue) presented in M-biased stimuli (*r* = 0.218, FDR adjusted *p* = 0.023). Conversely, participants with high trait anxiety showed *impaired* accuracy for recognizing direct-gaze fear (e.g., ambiguous threat cues) in P-biased stimuli (*r* = −0.220, FDR adjusted *p* = 0.023).

We next examined the effects of trait anxiety on the perception of neutral faces. Note that the congruent cue combination is different for neutral than for fearful faces. Direct-gaze is congruent with neutral faces, as direct-gaze increases the tendency of neutral faces to be perceived as neutral, whereas averted gaze causes neutral faces to be perceived as being somewhat fearful [e.g.,^25,29^]. For direct-gaze neutral faces (clear neutral, Fig. 2A) presented in M-biased stimulus, we observed that the participants with higher trait anxiety showed faster mean RTs (Fig. 2D, *r* = −0.229, FDR adjusted *p* = 0.034) and faster median RTs as well (trending, although not significant, *r* = −0.168, FDR adjusted *p* = 0.168). For averted-gaze neutral faces (ambiguous neutral, Fig. 2A) presented in P-biased stimulus, however, did not show significant trend (Fig. 2D, FDR adjusted *p* = 0.993 for mean RT and FDR adjusted *p* = 0.679).

As for the accuracy, we again found differential modulation by trait anxiety in recognition of clear cues vs. ambiguous cues in neutral faces (Fig. 2E). Participants with higher trait anxiety showed more accurate recognition of direct-gaze neutral faces (clear neutral) presented in M-biased stimulus (*r* = 0.196, FDR adjusted *p* = 0.042). Conversely, participants with higher trait anxiety made more error responses for averted-gaze neutral faces (ambiguous neutral) presented in P-biased stimulus (*r* = −0.233, *p* = 0.03). While clear, congruent cue combinations of eye gaze direction and facial expression are opposite for fearful vs. neutral faces, our results taken together already suggest that observers’ trait anxiety facilitates magnocellular processing of clear facial cues (averted-gaze fear and direct-gaze neutral), but impairs parvocellular processing of ambiguous facial cues.

We next wanted to ensure that this facilitated magnocellular processing and impaired parvocellular processing in participants with higher trait anxiety was specific to clear and ambiguous cues, respectively. We examined the correlation between the RTs and accuracy for ambiguous cues (e.g., direct-gaze fearful and averted-gaze neutral faces) presented in M-biased stimuli and for clear cues (e.g., averted-gaze fear and direct-gaze neutral faces) presented in P-biased stimuli. As shown in Supplementary Results 1, we only found significant negative correlations between trait anxiety and RT for averted-gaze fear (clear threat; *r* = −0.229 and FDR adjusted *p* = 0.028) presented in P-biased stimuli and direct-gaze neutral (clear neutral; *r* = −0.211 and FDR adjusted *p* = 0.028) presented in P-biased stimuli. Other trends, however, were not significant (all FDR adjusted *p’s* > 0.216). Therefore, we conclude that participants’ trait anxiety plays a specific role in modulating RTs and recognition accuracy for clear cues via magnocellular pathway and for ambiguous cues via parvocellular pathway.

Together, our behavioral results show that when clear cue combinations are presented (averted-gaze fearful or direct-gaze neutral faces) in the M-biased stimuli, increased trait anxiety had the effect of reducing RTs and facilitating recognition accuracy. Conversely, when ambiguous cue combinations (direct-gaze fearful and averted-gaze neutral faces) are presented in the P-biased stimuli, recognition accuracy decreased with higher trait anxiety, although it did not reduce the RTs. These results suggest that trait anxiety plays enhances magnocellular processing of clear, congruent facial cues and impairs parvocellular processing of ambiguous, incongruent facial cues.

### fMRI results

**Figure 3:**
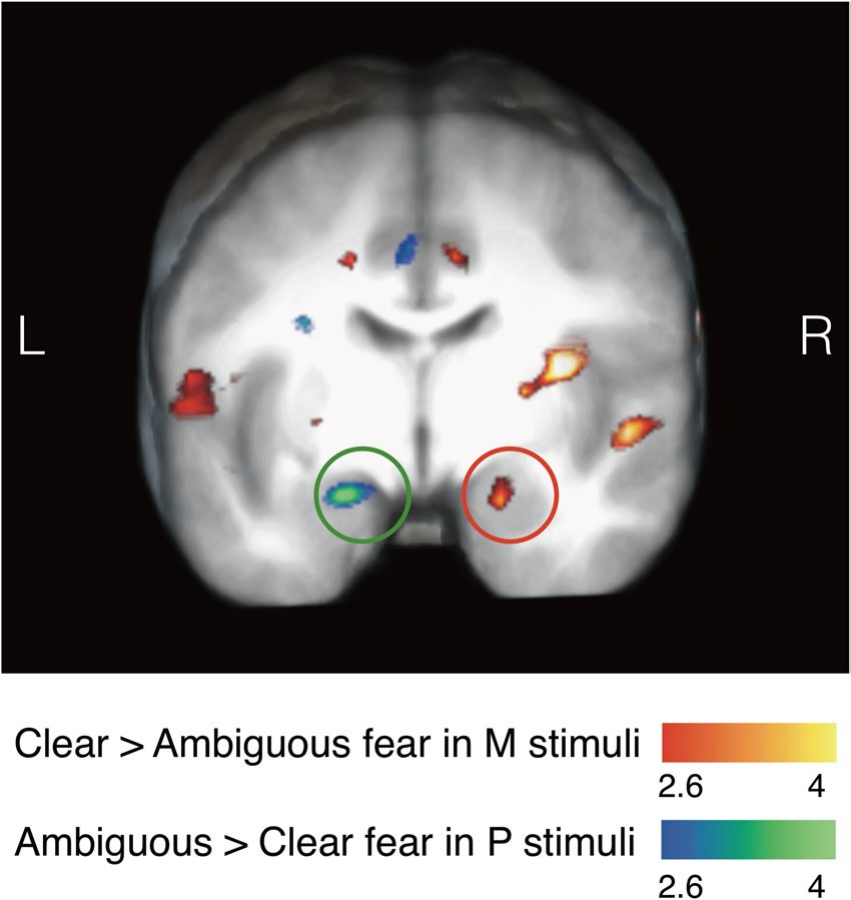
Activation map (*p* < 0.001, k = 5) corresponds to whole-brain analyses showing the left and right amygdalae activations for Ambiguous (direct-gaze fear) minus Clear (averted-gaze fear) threat cues presented in P-biased stimuli (blue-green) and for Clear (averted-gaze fear) minus Ambiguous (direct-gaze fear) threat cues presented in M-biased stimuli (red-yellow), respectively.

Table 1 presents the full list of activations (threshold: *p* < 0.05, FWE corrected; with the cluster defining threshold of *p* < 0.001, k = 10) for the contrasts of our primary interest: (1) Clear fear in M-biased stimulus – Ambiguous fear in M-biased stimulus, (2) Ambiguous fear in M-biased stimulus – Clear fear in M-biased stimulus, (3) Ambiguous fear in P-biased stimulus – Clear fear in P-biased stimulus, and (4) Clear fear in P-biased stimulus – Ambiguous fear in P-biased stimulus. We also report the result table with different threshold (*p* < 0.001, uncorrected) in Supplementary Results 2, in order to confirm that our results are robust across different thresholds. Previous studies have reported that the left and right amygdala showed differential preferences for clear threat cue (e.g., averted-gaze fear) vs. ambiguous threat cue (e.g., direct-gaze fear) and for M-biased vs. P- biased stimuli^23,31^. We replicated these findings by showing that the right amygdala was preferentially activated by clear threat (averted-gaze fear) in M-biased stimulus, whereas the left amygdala was preferentially activated by ambiguous threat (direct-gaze fear) in P-biased stimulus, as highlighted in Fig. 3.

**Table 1:** BOLD activations from group analysis, thresholded at *p* < 0.05, FWE-corrected (Cluster-defining threshold: *p* < 0.001, k = 10).

Contrast Name	Region label	Extent	t-value	MNI Coordinates		
				x	y	z
***M Clear - M Ambiguous***	R dorsolateral prefrontal cortex	272	4.34	57	41	4
	R anterior orbitofrontal cortex	272	3.921	18	56	−18
	L anterior orbitofrontal cortex	819	4.07	−21	56	−14
	R preSMA	238	3.97	12	29	50
	L middle temporal gyrus	421	3.76	−63	2	−30
	R hippocampus	266	3.63	33	−22	−14
	R amygdala	—	3.126	30	−2	−20
***M Ambiguous - M Clear***	None					
***P Ambiguous - P Clear***	L inferior temporal gyrus	536	5.720	−45	−37	−26
	L fusiform gyrus	536	4.241	−39	−73	−18
		—	4.177	−30	−52	−20
	L amygdala*	8	3.539	−18	2	−18
***P Clear - P Ambiguous***	None					

— indicates that this cluster is part of a larger cluster immediately above.

* indicates uncorrected *p* < 0.001, k = 5.

**Figure 4:**
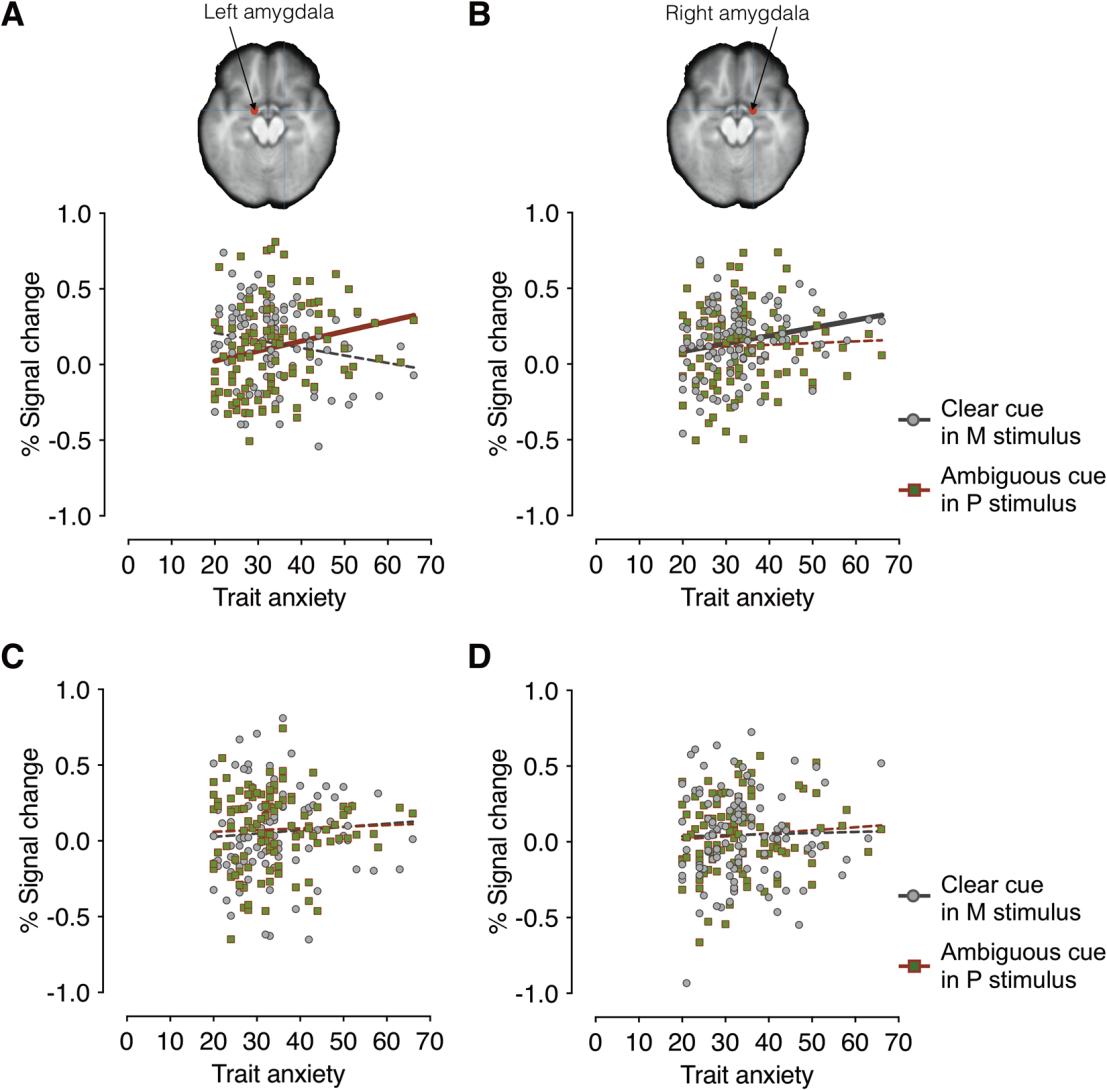
The left and right amygdala activation during perception of fearful (**A** and **B**) and neutral faces (**C** and **D**). (**A**) The scatter plot of the trait anxiety and the % signal change in the left amygdala when participants viewed clear fear (averted-gaze fear) faces presented in M-biased (in gray dots) and ambiguous fear (direct-gaze fear) faces in P-biased (in red-green dots) stimuli. The gray and red lines indicate the linear relationship between trait anxiety and the left amygdala activation for the M-biased stimuli and the P-biased stimuli, respectively. The solid, thicker lines indicate statistically significant correlations (FDR adjusted *p* < 0.05), whereas broken, thinner lines indicate that correlations were not statistically significant. (**B**) The scatter plot of the trait anxiety and the % signal change in the left amygdala when participants viewed clear fear (averted-gaze fear) faces presented in M-biased (in gray dots) and ambiguous fear (direct-gaze fear) faces presented in P-biased (in red-green dots) stimuli. (**C**) The scatter plot of the trait anxiety and the % signal change in the left amygdala when participants viewed clear neutral (direct-gaze neutral) faces presented in M-biased (in gray dots) and ambiguous neutral (averted-gaze neutral) faces presented in P-biased (in red-green dots) stimuli. (**D**) The scatter plot of the trait anxiety and the % signal change in the right amygdala when participants viewed clear neutral (direct-gaze neutral) faces presented in M-biased (in gray dots) and ambiguous neutral (averted-gaze neutral) faces presented in P-biased (in red-green dots) stimuli.

We next examined the partial correlation coefficients (r) between the participants’ trait anxiety and the left and right amygdala activations when the participants viewed averted-gaze fear (clear threat) presented in M-biased stimuli and direct-gaze fear (ambiguous threat) presented in P-biased stimuli (Fig. 4A and B). Observers’ trait anxiety showed marginally significant positive correlation with the activity in the left amygdala to direct-gaze fear faces (ambiguous threat) in P-biased stimuli (Fig. 4A; *r* = 0.196, FDR adjusted *p* = 0.060), but not to averted-gaze fear faces (clear threat) in M-biased stimuli (*r* = −0.174, FDR adjusted *p* = 0.085). Although both correlations were marginally significant, further comparison of the two correlation coefficients using the Fisher r-to-z transformation also confirmed that the correlation between trait anxiety and the left amygdala reactivity to direct-gaze fear faces in P-biased stimuli (r = 0.196, n = 108) was significantly greater than the correlation between trait anxiety and the left amygdala reactivity to averted-gaze fear faces in M-biased stimuli (*z* = 2.71, *p* = 0.006, two-tailed).

Conversely, we found that the trait anxiety positively correlated with the right amygdala responses to averted-gaze fear faces (clear threat) in M-biased stimuli (Fig. 4B; r = 0.234, FDR adjusted *p* = 0.028), but not to direct-gaze fear faces (ambiguous threat) in P-biased stimuli (*r* = −0.007, FDR adjusted *p* = 0.947). These findings suggest that modulation by observers’ trait anxiety was selective depending on the emotional valence, eye gaze, and pathway biases, highly lateralized in amygdala activation: High trait anxiety was associated with the increased right amygdala activation for magnocellular processing of averted-faze fearful faces (clear threat) and the increased left amygdala activation for parvocellular processing of direct-gaze fearful faces (ambiguous threat). Unlike for fearful faces, however, we did not observe any significant correlations between trait anxiety and the amygdala responses to neutral faces (Fig. 4C and D; all FDR adjusted *p’s* > 0.410).

**Figure 5:**
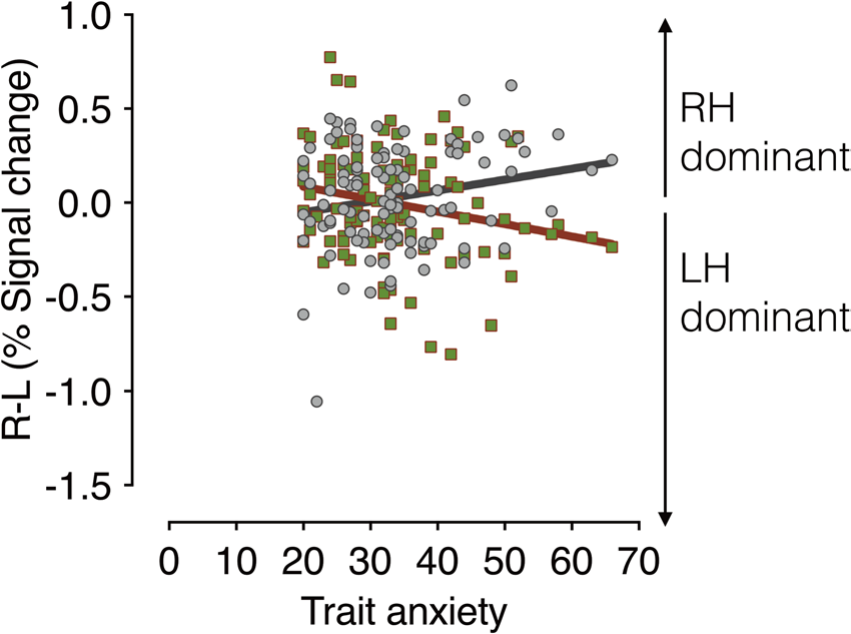
The magnitude of hemispheric lateralization in amygdala, obtained by subtracting the % signal change in the left amygdala from that in the right amygdala for each participant. The magnitude of hemispheric lateralization in amygdala when participants viewed clear fear (averted-gaze fear) faces presented in M-biased (dots in gray) and ambiguous fear (direct-gaze fear) faces presented in P-biased (dots in red-green) are plotted as a function of observers’ trait anxiety. The positive values indicate greater activation in the right amygdala (right hemisphere (RH) dominant) whereas the negative values indicate greater activation in the left amygdala (left hemisphere (LH) dominant). The gray and red lines indicate the linear regressions with trait anxiety for the M-biased and P-biased stimuli, respectively. Solid, thick lines indicate that correlations were statistically significant (FDR adjusted *p* < 0.05).

Because previous studies have suggested differential preference of the left and right amygdalae for P-biased direct fear and M-biased averted fear^23,31^, we next examined the magnitude of the hemispheric asymmetry by subtracting the left amygdala activation from the right amygdala activation for each condition. We plotted the difference between the activation levels in the left and the right amygdala as a function of the participants’ trait anxiety (Fig. 5). Positive values indicate right amygdala activation greater than the left amygdala (e.g., right hemisphere (RH) dominant), whereas negative values indicate left amygdala activation greater than the right amygdala (e.g., left hemisphere (LH) dominant). While we did not observe significant lateralization for the neutral faces (all FDR adjusted *p’s* > 0.198, Supplementary Results 3), we found the significant lateralization for the fearful faces, systematically modulated by trait anxiety. The right amygdala became more dominant over the left amygdala with increasing trait anxiety for averted-gaze fear (clear threat) presented in the M-biased stimulus (*r* = 0.289, FDR adjusted *p* = 0.004). Conversely, the left amygdala became more dominant over the right amygdala with the increasing trait anxiety for direct-gaze fear (ambiguous threat) presented in the P-biased stimulus (*r* = −0.236, FDR adjusted *p* = 0.013). These results suggest that observers with higher trait anxiety show more pronounced hemispheric lateralization between the left and right amygdala, with the right amygdala being more dominant for magnocellular processing of clear threat (averted-gaze fear) and the left amygdala being more dominant for the parvocellular processing of ambiguous threat (direct-gaze fear).

## Discussion

Anxiety can be hugely disruptive to everyday life, and over a quarter of the population suffers from an anxiety disorder during their lifetime^64^. The primary goal of this study was to examine how individuals’ trait anxiety modulates behavioral and amygdala responses in reading compound facial threat cues - one of the most common and important social stimuli - via two major visual streams, the magnocellular and parvocellular pathways. Here we employed a larger (N = 108) and more representative (age range 20–70) community sample than in most of the previous studies examining the effects of anxiety on the affective facial processing^29,49,54^. We report three main findings: 1) Compared to low trait-anxiety individuals, high trait-anxiety individuals were more accurate for M-biased averted-gaze fear (a clear threat cue combination), but less accurate for P- biased direct-gaze fear (ambiguous threat cues). The opposite was true for neutral face stimuli such that participants were better at correctly recognizing M-biased direct-gaze neutral faces, but less accurate for P-biased averted-gaze neutral faces; 2) increased right amygdala reactivity was associated with higher trait anxiety only for M-biased averted- gaze fear (clear threat stimuli), whereas increased left amygdala reactivity was associated with higher trait anxiety only for P-biased direct-gaze fear (ambiguous threat stimuli), and 3) the magnitude of this laterality effect for the fearful faces increased with higher trait anxiety. The current findings indicate differential effects of observers’ anxiety on threat perception, resulting in facilitated magnocellular processing of clear facial threat cues (associated with the increased right amygdala reactivity) and impaired parvocellular processing of ambiguous facial threat cues (associated with the increased left amygdala reactivity). Since the current study was limited to correlation and linear regression analyses, inferring causal architecture and interplay between these variables is not possible. Future studies will be needed to characterize the causality and directionality of the interplay between trait anxiety, amygdala activations, and behavioral measurements, perhaps by employing dynamic causal modeling or psychophysiological interactions analyses.

Previous studies^12,65^ and cognitive formulations of anxiety^66,67^ have suggested that vulnerability to anxiety is associated with increased vigilance for threat-related information in general (for review, see^68^). However, trait anxiety appears to play a more specific role in dynamically regulating behavioral and neural responses to threat displays, depending on which visual pathway is engaged and on clarity or ambiguity of the threat. Our findings of the enhanced responses to averted-gaze fear (clear threat) in the high trait-anxiety individuals are largely consistent with previous work on the effect of anxiety on perception of fearful faces. For example, observing averted- gaze fear resulted in enhanced integrative processing and cuing effects for those with high (but not low) trait anxiety^11,29,55–57^. What was not clear from the previous work, however, was the differential effect of anxiety when fearful faces contained direct eye gaze (ambiguous threat cue), compared to averted eye gaze, and the contribution of the main visual pathways (M and P) to this processing. The achromatic M-pathway has characteristics that make it well suited to rapid processing of coarse, ‘gist’ information (see^69^ for a review), and has been implicated in triggering top-down facilitation in object recognition in the orbitofrontal cortex^58,70^, and recognition of clear threat in scene images^59,60^.

Here we observed selective facilitation in recognizing M-biased averted-gaze fearful faces (e.g., rapid detection of clear threat cues), but also selective impairment in recognizing P- biased direct-gaze fearful faces (e.g., detailed analysis of ambiguous threat cues) in high trait-anxiety individuals. This finding suggests that the effect of higher trait anxiety may be to increase responsiveness to, and facilitate reflexive processing of, clear threat-related cues (e.g., averted-gaze fear). However it can be also disruptive when threat cues require detailed, reflective processing to resolve ambiguity (e.g., P-biased processing of threat ambiguity). While recognition accuracy of ambiguous facial cues (direct-gaze fear and averted-gaze neutral faces) was similar for M- and P-biased faces with low trait-anxiety, the accuracy decreased for the P-biased stimuli with anxiety.

To support and extend our behavioral findings, we also observed that high trait-anxiety individuals showed increased amygdala reactivity in a specific manner, such that the right amygdala activity increased along with anxiety only to the M-biased averted-gaze fear faces, and the left amygdala activity became greater only to the P-biased direct-gaze fear as trait anxiety increased. The effect of anxiety on amygdala attunement in the interaction of emotional valence, eye gaze direction, and pathway biasing was particularly pronounced for fearful faces but not for neutral faces, suggesting that this modulation by anxiety is specifically associated with threat detection from face stimuli. Consistent with this, we did not find any evidence for this modulation by anxiety in the fusiform face area (FFA; Supplementary Results 4).

The existing literature on the laterality effect on emotional processing in high vs. low anxiety individuals is rather mixed. Many of the studies have shown increased right hemisphere (RH) dominance in affective processing of high-anxiety individuals. For example, a left visual field (LVF) bias has been reported for processing fearful faces in high-anxiety individuals^13^ and masked angry faces presented only in LVF captured more attentional resources in high-anxiety individuals^53^. Nonclinical state anxiety was also found to be associated with increased right-hemisphere activity, as measured by regional blood flow^71^, suggesting that sensitivity of right hemisphere to the presence of threat stimuli seems to be especially, although not exclusively, heightened in high-anxiety individuals. However, there are also some studies showing the association between trait anxiety and the left hemisphere (LH) activation^72,73^ and larger-anxiety-related attentional bias for threatening faces presented in RVF, relative to the LVF^12,50^. Here, we directly tested for laterality effects in the left and right amygdala and found that trait anxiety modulates amygdala reactivity in both hemispheres, but with different processing emphases, such that high trait anxiety was associated with increased right amygdala reactivity to M-biased averted-gaze fear (clear threat), but increased left amygdala reactivity to P-biased direct-gaze fear (ambiguous threat). This result is also in line with the notion that reflective threat perception (e.g., resolving ambiguity from direct-gaze fear) is more left-lateralized, whereas reflexive processing (e.g., detecting clear threat from averted-gaze fear) is more right-lateralized^23,31,62,63^. Furthermore, such hemispheric lateralization became more pronounced in participants with higher trait anxiety. Thus, trait anxiety appears to play an important role in regulating the balance between the left and right amygdala responses depending on types of threat processing and pathway-biasing.

To conclude, the current study provides the first behavioral and neural evidence that trait anxiety differentially modulates the magnocellular processing of clear emotional cues (e.g., congruent combination of facial cues: averted-gaze fear and direct-gaze neutral) and parvocellular processing of ambiguous emotional cues (e.g., direct-gaze fear and averted gaze neutral). Observers’ trait anxiety also plays a specific role in differentially shaping the hemispheric lateralization in the amygdala reactivity, as a result of the complex, but systematic interplay of cue ambiguity and the visual pathway biases. Using a larger and more representative sample of a population (N = 108), the current findings on the differential effects of trait anxiety on the information processing via M- vs. P-pathways and the hemispheric lateralization provide a more generalizable model for neurocognitive mechanisms underlying the perception of facial threat cues and its systematic modulation by anxiety.

## Method

### Participants

108 participants (65 female) from the Massachusetts General Hospital (MGH) and surrounding communities participated in this study. The age of the participants ranged from 18 to 70 (mean = 37.05, SD = 14.7). The breakdown of participants’ ethnic background is detailed in Supplementary Table 1. All had normal or corrected-to-normal visual acuity and normal color vision, as verified by the Snellen chart^74^, the Mars letter contrast sensitivity test^75^, and the Ishihara color plates^76^. Informed consent was obtained from the participants in accordance with the Declaration of Helsinki. The experimental protocol was approved by the Institutional Review Board of MGH, and all experiments were performed in accordance with the guidelines and regulations prescribed by the committee of Institutional Review Board at MGH, Boston, Massachusetts. The participants were compensated with $50 for their participation in this study.

### Apparatus and stimuli

The stimuli were generated using MATLAB (Mathworks Inc., Natick, MA), together with the Psychophysics Toolbox extensions^77,78^. The stimuli consisted of a face image presented in the center of a gray screen, subtending 5.79° × 6.78° of visual angle. We utilized a total of 24 face identities (12 female), 8 identities selected from the Pictures of Facial Affect^79^, 8 identities from the NimStim Emotional Face Stimuli database^80^, and the other 8 identities from the FACE database^81^. The face images displayed either a neutral or fearful expression with either a direct gaze or averted gaze, and were presented as M-biased, P-biased, or Unbiased stimuli, making 288 unique visual stimuli in the end. Faces with an averted gaze had the eyes pointing either leftward or rightward.

Each face image was first converted to a two-tone image (black-white; termed the Unbiased stimuli from here on). From the two-tone image, low-luminance contrast (<5% Weber contrast), achromatic, grayscale stimuli (magnocellular-biased stimuli), and chromatically defined, isoluminant stimuli (red-green; parvocellular-biased stimuli) were generated. The low-luminance contrast images were designed to preferentially engage the M-pathway, while the isochromatic images were designed to engage the P-pathway, as such image manipulation has been employed successfully in previous studies^58,61,82–87^. The foreground-background luminance contrast for achromatic M-biased stimuli and the isoluminance values for chromatic P-biased stimuli vary somewhat across individual observers. Therefore, these values were established for each participant in separate test sessions, with the participant positioned in the scanner, before commencing functional scanning. This ensured that the exact viewing conditions were subsequently used during functional scanning in the main experiment. Following the procedure in Kveraga *et al*.^58^, Thomas *et al*.^61^, and Boshyan *et al*.^59^, the overall stimulus brightness was kept lower for M stimuli (the average value of 115.88 on the scale of 0–255) than for P stimuli (146.06) to ensure that any processing advantages for M-biased stimuli were not due to greater overall brightness of the M stimuli, as described in detail below.

### Procedure

Before the fMRI session, participants completed the Spielberger State-Trait Anxiety Inventory (STAI;^88^). Participants were then positioned in the fMRI scanner and asked to complete the two pretests to specify the luminance values for M stimuli and chromatic values for P stimuli and the main experiment. The visual stimuli containing a face image were rear-projected onto a mirror attached to a 32-channel head coil in the fMRI scanner, located in a dimly lit room. The following procedures for pretests used to establish the isoluminance point and the appropriate luminance contrast are standard techniques and have been successfully used in many studies exploring the M- and P-pathway contributions to object and scene recognition, visual search, schizophrenia, dyslexia, and simultanagnosia^58–61,82–87^.

### Pretest 1: Measuring luminance threshold for M-biased stimuli

The appropriate luminance contrast was determined by finding the luminance threshold via a multiple staircase procedure. Figure 1A illustrates a sample trial of Pretest 1. Participants were presented with visual stimuli for 500 msec and instructed to make a key press to indicate the facial expression of the face that had been presented. They were required to choose one of the four options: 1) neutral, 2) angry, 3) fearful, or 4) did not recognize the image. One-fourth of the trials were catch trials in which the stimulus did not appear. To find the threshold for foreground-background luminance contrast, our algorithm computed the mean of the turnaround points above and below the gray background ([120 120 120] RGB value on the 8-bit scale of 0–255). From this threshold, the appropriate luminance (~3.5% Weber contrast) value was computed for the face images to be used in the low-luminance-contrast (M-biased) condition. As a result, the average foreground RGB values for M-biased stimuli were [116.5(±0.2) 116.5(±0.2) 116.5(±0.2)].

### Pretest 2: Measuring red-green isoluminance value for P-biased stimuli

For the chromatically defined, isoluminant (P-biased) stimuli, each participant’s isoluminance point was determined using heterochromatic flicker photometry with two- tone face images displayed in rapidly alternating colors, between red and green. The alternation frequency was ~14 Hz, because in our previous studies^58–61^ we obtained the best estimates for the isoluminance point (e.g., narrow range within-subjects and low variability between-subjects;^58^) at this frequency. The isoluminance point was defined as the color values at which the flicker caused by luminance differences between red and green colors disappeared and the two alternating colors fused, making the image look steady. On each trial (Fig. 1B), participants were required to report via a key press whether the stimulus appeared flickering or steady. Depending on the participant’s response, the value of the red gun in [r g b] was adjusted up or down in a pseudorandom manner for the next cycle. The average of the values in the narrow range when a participant reported a steady stimulus became the isoluminance value for the subject used in the experiment. Thus, isoluminant stimuli were defined only by chromatic contrast between foreground and background, which appeared equally bright to the observer. The average foreground red value was 151.7(±5.35) on the background with green value of 140.

### Main experiment

Figure 1C illustrates a sample trial of the main experiment. After a variable pre-stimulus fixation period (200–400 msec), a face stimulus was presented for 1000 msec, followed by a blank screen (1100–1300 msec). Participants were required to indicate whether a face image looked fearful or neutral, as quickly as possible. Key-target mapping was counterbalanced across participants: One half of the participants pressed the left key for neutral and the right key for fearful and the other half pressed the left key for fearful and the right key for neutral. Feedback was provided on every trial.

### fMRI data acquisition and analysis

fMRI images of brain activity were acquired using a 1.5 T scanner (Siemens Avanto) with a 32-channel head coil. High-resolution anatomical MRI data were acquired using T1-weighted images for the reconstruction of each subject’s cortical surface (TR = 2300 ms, TE = 2.28 ms, flip angle = 8°, FoV = 256 × 256 mm^2^, slice thickness = 1 mm, sagittal orientation). The functional scans were acquired using simultaneous multislice, gradient- echo echoplanar imaging with a TR of 2500 ms, three echoes with TEs of 15 ms, 33.83 ms, and 52.66 ms, flip angle of 90°, and 58 interleaved slices (3 × 3 × 2 mm resolution).

Scanning parameters were optimized by manual shimming of the gradients to fit the brain anatomy of each subject, and tilting the slice prescription anteriorly 20–30° up from the AC-PC line as described in the previous studies^58,89,90^, to improve signal and minimize susceptibility artifacts in the subcortical brain regions. For each participant, the first 15 seconds of each run were discarded, followed by the actual acquisition of 96 functional volumes per run (lasting 4 minutes). There were four successive functional runs, providing the 384 functional volumes per subject in total, including the 96 null, fixation trials and the 288 stimulus trials. In our 2 (emotion: fear vs. neutral) × 2 (eye gaze direction: direct vs. averted) × 3 (bias: unbiased, M-biased, and P-biased) design, each condition had 24 repetitions, and the sequence of total 384 trials was optimized for hemodynamic response estimation efficiency using the optseq. 2 software (https://surfer.nmr.mgh.harvard.edu/optseq/).

The acquired functional images were pre-processed using SPM8 (Wellcome Department of Cognitive Neurology). The functional images were corrected for differences in slice timing, realigned, corrected for movement-related artifacts, coregistered with each participant’s anatomical data, normalized to the Montreal Neurological Institute (MNI) template, and spatially smoothed using an isotropic 8-mm full width half-maximum (FWHM) Gaussian kernel. Outliers due to movement or signal from preprocessed files, using thresholds of 3 SD from the mean, 0.75 mm for translation and 0.02 radians rotation, were removed from the data sets, using the ArtRepair software^91^.

Subject-specific contrasts were estimated using a fixed-effects model. These contrast images were used to obtain subject-specific estimates for each effect. For group analysis, these estimates were then entered into a second-level analysis treating participants as a random effect, using one-sample t-tests at each voxel. Age and gender of participants were used as covariates to be controlled, in order to assess the effect of anxiety. Because our recent findings^23,33,92^ have suggested that clear facial cues (e.g., averted fearful faces) are predominantly processed via magnocellular pathway whereas ambiguous facial cues (e.g., direct gazed fearful faces) are predominantly processed via parvocellular pathway, we examined differences in brain activations for following contrasts as our main interests: [Clear fear in M stimuli – Ambiguous fear in M stimuli], [Ambiguous fear in M stimuli – Clear fear in M stimuli], [Ambiguous fear in P stimuli – Clear fear in P stimuli], and [Clear fear in P stimuli – Ambiguous fear in P stimuli]. For illustration purposes, the group contrast images were overlaid onto a group average brain using MRIcroGL software (http://www.mccauslandcenter.sc.edu/mricrogl/home). As shown in Table 1, brain activations above the threshold of *p* < 0.05 (FWE-corrected for multiple comparison, with *p* < 0.001 and k = 10 for cluster-defining threshold) were reported for these contrasts.

For ROI analyses, we selected a contrast between all the visual stimulation trials and baseline (e.g., Null trials). From this contrast, we used the rfxplot toolbox (http://rfxplot.sourceforge.net) for SPM and extracted the beta weights from the left and right amygdala for the two conditions of our main interest: Clear threat cue (averted-gaze fear) in M stimuli and Ambiguous threat cue (direct-gaze fear) in P stimuli. We used the same MNI coordinates for the left and right amygdala (x =  ± 18, y = −2, y = 16) as reported in the relevant previous work on the role of anxiety in perceiving fear faces with direct or averted eye gaze^29^. Around these coordinates, we defined 6mm spheres and extracted all the voxels from each individual participant’s functional data within those spheres. The extracted beta weights for each of the two conditions were subjected to a linear regression and correlation analyses along with the participants’ trait anxiety scores. For each of the correlation results, we report correlation coefficient (*r*) values and FDR adjusted *p* values.

### Data availability

The datasets generated and/or analyzed during the current study are available from H.Y.I. or the corresponding author on reasonable request.

## Electronic supplementary material

Supplementary Information
