# Supplementary material for: Observer’s anxiety facilitates magnocellular processing of clear facial threat cues, but impairs parvocellular processing of ambiguous facial threat cues
Source: Sci Rep. 2017 Nov 9;7:15151. doi: 10.1038/s41598-017-15495-2 (PMC5680327; doi:10.1038/s41598-017-15495-2)
Supplement: Supplementary file 1 — Supplementary Information [file 41598_2017_15495_MOESM1_ESM.pdf]

Supplementary Information

Observer's anxiety facilitates magnocellular processing of clear facial threat cues, but impairs parvocellular processing of ambiguous facial threat cues.

Hee Yeon Im[1,2], Reginald B. Adams, Jr.[3], Jasmine Boshyan[1,2], Noreen Ward[1], Cody A. Cushing[1], Kestutis Kveraga[1,2]

[1]Athinoula A. Martinos Center, Department of Radiology, Massachusetts General Hospital, Charlestown, MA, U.S.A.

[2]Department of Radiology, Harvard Medical School, Boston, MA, U.S.A.

[3]Department of Psychology, The Pennsylvania State University, State College, PA, U.S.A.

Supplementary Results 1. Behavioral results (response time and proportion correct) for ambiguous threat cues (direct-gaze fear faces) presented in M-biased stimuli and for clear threat cues (averted-gaze fear faces) presented in P-biased stimuli.

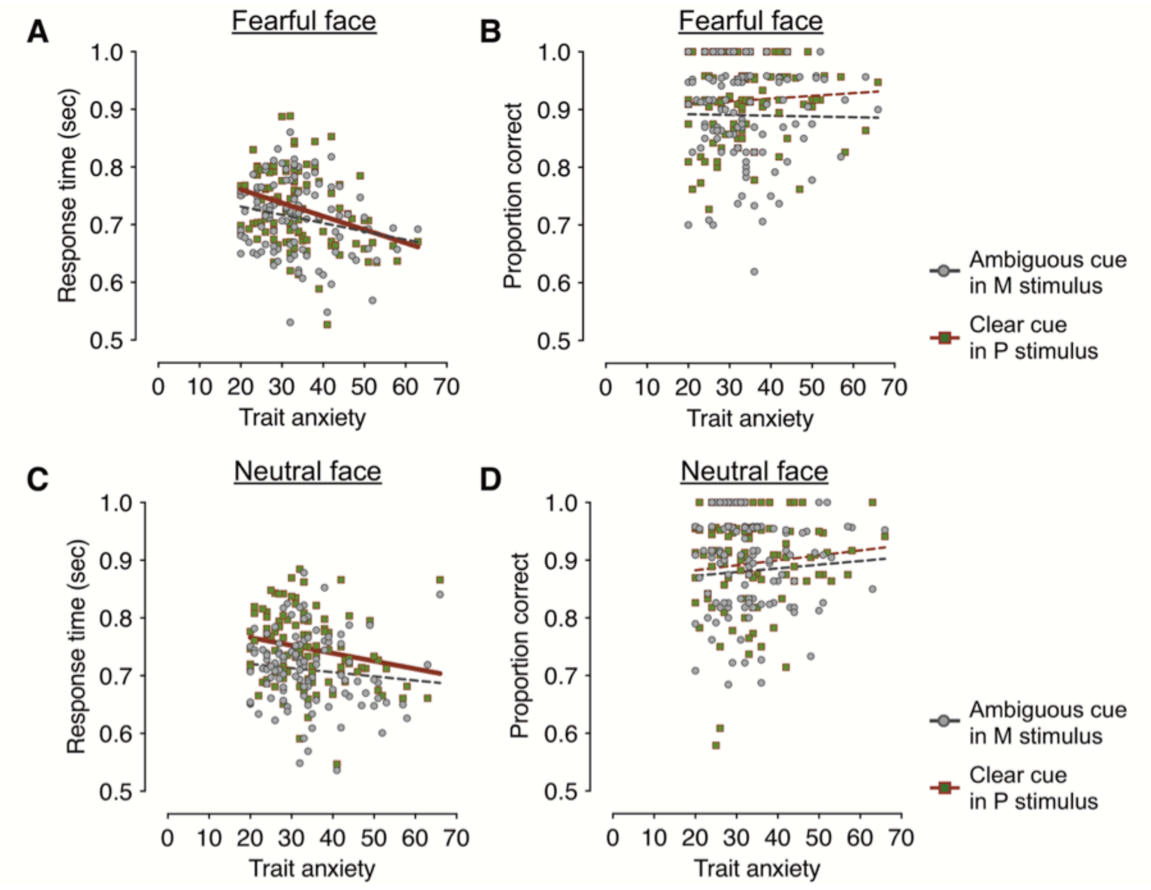

Supplementary Results 2. BOLD activations from group analysis, thresholded at *p* < 0.001, uncorrected, k > 5.

Contrast Name				MNI Coordinates		
	Region Label	Extent	t-value	x	y	z
M averted fear - M direct fear	R dorsolateral prefrontal cortex	83	4.297	57	41	4
	L anterior orbitofrontal cortex	27	4.059	-21	56	-14
	R anterior orbitofrontal cortex	37	3.945	18	56	-18
		9	3.502	36	50	-18
	L preSMA	13	3.713	-9	29	46
	R preSMA	100	3.985	12	29	50
	L precental gyrus	20	3.519	-51	2	44
	L precentral gyrus	7	3.294	-36	-1	48
	R posterior cingulate cortex	9	3.937	15	-31	40
	L middle temporal gyrus	9	3.749	-63	2	-30
	R middle temporal gyrus	37	3.602	72	-43	-12
	R lateral orbitofrontal cortex	31	3.741	39	26	-6
	L insula	26	3.738	-21	23	0
	R hippocampus	37	3.617	33	-22	-14
	R superior frontal gyrus	48	3.583	39	2	42
	L thalamus	10	3.568	-6	-19	6
	L occipital cortex (BA18)	33	3.449	-24	-94	-12
	L middle cingulate cortex	9	3.436	-12	-13	38
	L intraparietal sulcus	7	3.413	-33	-52	40
	L supramarginal gyrus	6	3.237	-57	-31	40
	R superior parietal lobule	7	3.180	39	-52	50
	R amygdala *	55	2.904	27	2	-14
	L cerebellum	24	4.061	-9	-49	-16
		24	3.522	-6	-82	-32
		17	3.242	-30	-73	-48
	R cerebellum	19	3.477	9	-82	-36
M direct fear - M averted fear	None					
P direct fear - P averted fear	L inferior temporal gyrus	216	5.772	-45	-37	-26
		20	3.841	-60	-16	-32
	R inferior temporal gyrus	17	3.543	51	-43	-24
	L fusiform cortex	100	4.202	-39	-73	-18
	R fusiform cortex	18	3.793	36	-28	-34
	L superior frontal gyrus	37	3.729	-36	32	52
	R superior frontal gyrus	19	3.465	21	44	52
	R occipital cortex (BA19)	32	3.996	27	-82	-18
	R supplementary motor area	21	3.866	9	2	48
	L amygdala	11	3.531	-18	2	-20
	L brainstem	7	3.508	-3	-28	-4
	R retrosplenial cortex	22	3.458	6	-46	10
	R postcentral gyrus	16	3.428	57	-28	56
P averted fear - P direct fear	R anterior cingulate cortex	89	3.860	15	38	18
	L insula	19	3.527	-30	8	14

Supplementary Results 3. The magnitude of hemispheric lateralization in amygdala, obtained by subtracting the % signal change in the left amygdala from that in the right amygdala, when participants were viewing neutral faces. We did not find any significant trends between the magnitude of lateralization and trait anxiety, when observers were viewing neutral faces.

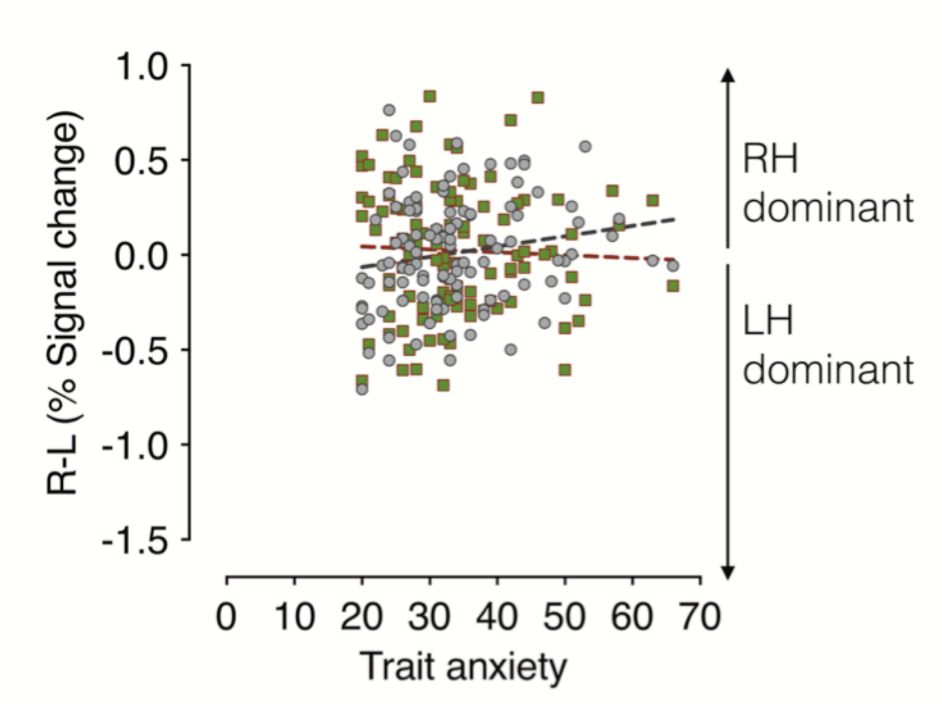

Supplementary Results 4. The left and right FFA [±42 -52 -26] activation during perception of fearful faces.

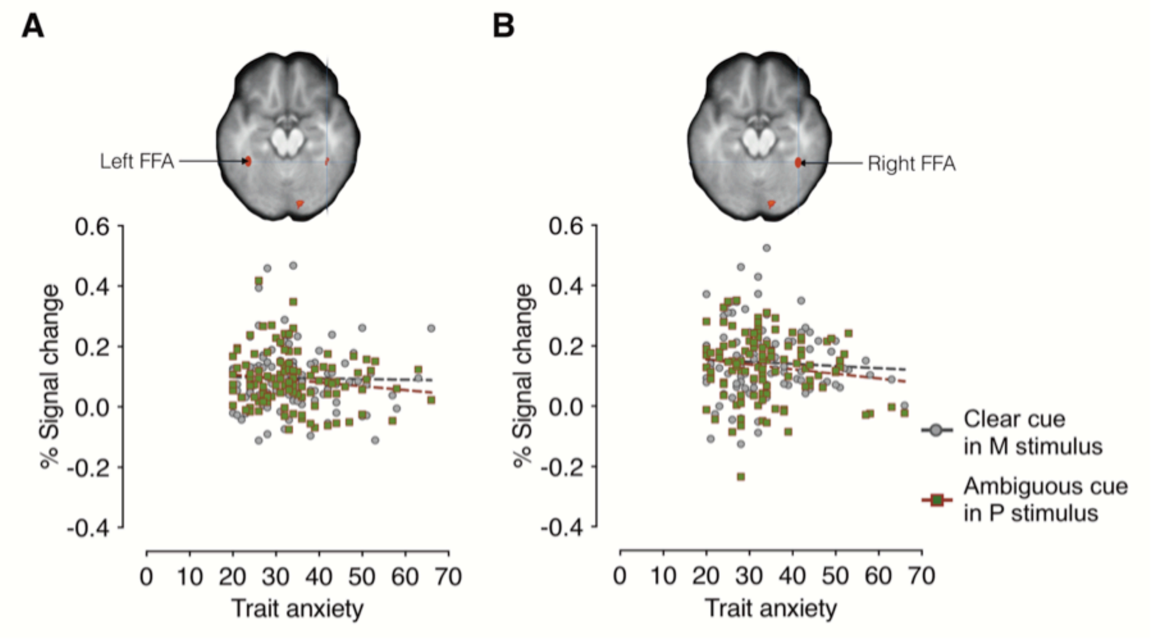

Supplementary Table 1. The breakdown of the participants' ethnic background.

Racial Categories	Not Hispanic or Latino	Hispanic or Latino	Unknown/Not reported	Total
Asian	24			
Black or African american	15			
White	54	7		
Total	93	7	8	108
